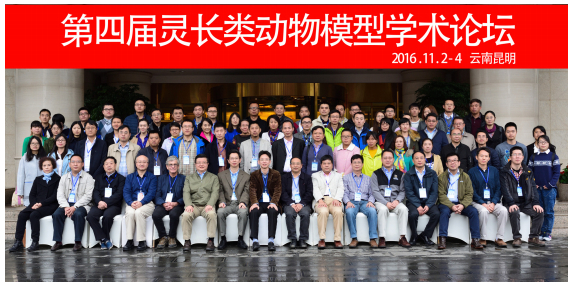# Meeting report: the 4^th^ symposium on animal models of non-human primates in Kunming, Yunnan, China

**DOI:** 10.13918/j.issn.2095-8137.2016.6.361

**Published:** 2016-11-18

**Authors:** Jia-Li LI, CHOU Shinn, Yong-Tang ZHENG, Xu-Dong ZHAO, Xin-Tian HU

**Affiliations:** ^1^Key Laboratory of Animal Models and Human Disease Mechanisms of the Chinese Academy of Sciences & Yunnan Province, Kunming Institute of Zoology, Chinese Academy of Sciences, Kunming 650223, China; ^2^Kunming Primate Research Center, Kunming Institute of Zoology, Chinese Academy of Sciences, Kunming 650223, China

From 2 to 4 November, 2016, the 4^th^ Symposium on Animal Models of Non-Human Primates (NHP) was held in Kunming, Yunnan, China. This meeting was organized by the Key Laboratory of Animal Models and Human Disease Mechanisms of the Chinese Academy of Sciences (CAS) & Yunnan Province, Kunming Primate Research Center (KPRC), *Zoological Research*, and Kunming Institute of Zoology (KIZ), CAS. 

The agenda was led by a keynote lecture from Dr. Yong-Gang Yao, the director of KIZ and chair of the symposium. Presentations from 18 invited speakers and eight local speakers from KIZ were delivered under four topic sessions: "Infectious Diseases and Vaccines", "Neuroscience and Neurological Disorders", "Stem Cell and Transgenic NHP Models" and "NHP Animal Welfare and Others". Another highlight of the symposium was the tour of the KPRC organized by Drs. Yong-Tang Zheng and Jia-Li Li. Since its establishment in 2005, the KPRC has become a highly-regarded NHP research and development center, serving as one of the major bases for biomedical research in China.

As emphasized in the symposium, NHP animal models are not only irreplaceable in basic research, but also of critical importance in a wide range of scientific and clinical investigations. The symposium, together with the previous series (Yao et al., 2015), also provided opportunities to promote communication and facilitate collaboration among researchers in the field of NHP studies, from basic research to translational medicine.

## KEYNOTE LECTURE

Following a warm welcome by **Dr. Yong-Tang Zheng** (KIZ, CAS), the co-chair of the symposium, Dr. Yong-Gang Yao (KIZ, CAS) delivered the keynote lecture on the establishment of the National Research Facility for Phenotypic and Genotypic Analysis of Model Animals (Primates) (NRFPGAMA) and its related research programs on phenotypic and genetic studies. In it, the blueprint of the NRFPGAMA was presented, which highlighted its involvement in sophisticated crosstalk from NHP breeding, NHP resource management, mega data collection and analysis, and relevant applications in various research fields and drug developments. Also discussed were the future directions of this major, multifaceted scientific facility, which will serve as much more than a traditional research platform. Its potential applicable purposes will be greatly extended, and it will continue to be actively involved in coordinating multi-disciplinary research and application in biomedical studies and drug development, as well as to lead the future primate related researches. 

## FEATURED TOPIC SESSIONS

### Infectious diseases and vaccines

**Dr. Zhi-Wei Chen** (AIDS Institute, University of Hong Kong) described his group's pioneering studies to elucidate the mucosal seeding of SARS-CoV infection using the Chinese macaque model (Liu et al., 2016).

**Dr. Xia Jin **(Institute Pasteur of Shanghai, CAS) presented research entitled "Dengue and Zika rhesus monkey model in the development of vaccines", in which the history and current applications of rhesus macaque (*Macaca mulatta*) models in infectious disease and vaccine evaluations of dengue and Zika viruses were introduced (Dudley et al., 2016; Liang et al., 2015; Logan, 2016). Dr. Jin also presented work on the development of subunit dengue vaccines and the use of NHP for the evaluation of vaccine conferred protection (Cheng et al., 2016; Jin et al., 2015;). 

**Dr. Xiao-Ping Chen** (Guangzhou Institutes of Biomedicine and Health, CAS) reported on a rhesus macaque model of co-infection with malaria and simian immunodeficiency virus (SIV), along with antiretroviral therapy (ART) treatment. Dr. Chen showed that *Plasmodium* infection reduced the replication-competent virus pool in resting CD4^+^ T cells (a major viral reservoir), which might be attributable to the activation and apoptosis of memory CD4^+^ T cells induced by malaria, with histone acetylation and NF-κB activation in resting CD4^+^ T cells also important in this reduction. Therefore, because more HIV-1-infected individuals in malaria-endemic areas are receiving ART treatment, clinicians should determine whether some patients co-infected with *Plasmodium* are experiencing virological benefits (Zhan et al., 2014). 

**Dr. Yong-Tang Zheng** (KIZ, CAS) delivered a presentation entitled "Basic biological research of northern pig-tailed macaques (*Macaca leonine*, PTMs) and its application in AIDS models". Dr. Zheng obtained 250 PTMs from Vietnam, and analyzed their hematology and blood chemistry parameters, immunoglobulin, complements and CRP levels, immune cells in the peripheral blood mononuclear cells (PBMCs), classical MHC class I genes, and pathogens. The physiological and biochemical indexes of PTMs suggested that, compared with Chinese rhesus macaques, PTMs are genetically closer to humans and thus might serve as a better NHP experimental animal (Lei et al., 2014; Lian et al., 2016; Pang et al., 2013; Zhang et al., 2014, 2016; Zheng et al., 2014; Zhu et al., 2015). Based on these findings, Dr. Zheng's team inoculated PTMs with HIV-1 strains and monitored the infections for three years. The results suggest that HIV-1-infected NPMs might function as a potential NHP animal model in HIV-1 latency studies and in developing novel therapeutic strategies.

**Dr. Bo Zhang **(Wuhan Institute of Virology, CAS) discussed their recent study on the isolation of the Chikungunya virus (CHIKV) and the establishment of its reverse genetics platform. CHIKV is a member of the *Alphavirus* genus. It is an important mosquito-borne human pathogen, which can cause abrupt high fever, headache, rashes, myalgia, and arthralgia, with arthritis possibly lasting for months or years. So far, however, no effective antiviral drugs or vaccines against CHIKV infection are available. Dr. Zhang's team recently isolated a CHIKV strain (GenBank Accession No. KC488650) from a clinically CHIKV-positive patient in China through serial passages in C6/36 cells, and found that it belonged to the Asian lineage of CHIKV. Using this isolated strain, an infectious clone of CHIKV and a reporter virus (eGFP-CHIKV) with an eGFP gene were constructed and used to investigate viral replication through measuring eGFP expression level. The eGFP-CHIKV reporter virus was then used to identify inhibitors and screen neutralizing antibodies against CHIKV (Deng et al., 2016).

**Dr. Ling Chen** (Guangzhou Institutes of Biomedicine and Health, CAS) discussed his group's work on the preparation of neutralized monoclonal antibodies (mAbs) using immunized macaques to against outbreaks of highly infectious diseases (Meng et al., 2013; Pan et al., 2014).

**Dr. Jian-Qing Xu** (Fudan University) presented work in the sequential immunization strategy of heterologous HIV immunogens (Zhou et al., 2016).

### Neuroscience and neurological disorders

**Dr. Bao-Ming Li** (Nanchang University) provided evidence that myelination of the prefrontal cortex (PFC) is essential for the development of projection neurons in the PFC. His group showed that adverse early-life experience, i.e., deprivation of parental relationships, can induce permanent phenotypic changes and impair the cognitive functions associated with PFC. Myelination is necessary for medial PFC (mPFC)-dependent behaviors. Blockade of oligodendrocyte (OL) differentiation or lysolecithin-induced demyelination can impair mPFC functions. Histone deacetylases 1/2 (HDAC1/2) were drastically reduced in neonatal maternal separation (NMS) rats; the inhibition of HDAC1/2 promoted Wnt activation, which negatively regulated OL development, whereas, the selective inhibition of Wnt signaling by XAV939 partly rescued myelination arrestment and behavior deficiency caused by NMS. These findings indicate that, to some extent, NMS impairs mPFC cognitive functions through modulation of oligodendrogenesis and myelination (Yang et al., 2016). 

**Dr. Xiao-Zhong Peng** (Chinese Academy of Medical Sciences) presented work on the roles of non-coding RNAs in neurodevelopmental disorders, and showed that microRNAs (miRNAs) in the development of brain and non-coding molecules might assist in the development and function of the central nervous system and drive neurodevelopmental disorders (Lin et al., 2016). 

**Dr. Xiao-Qing Zhang** (Tongji University) described the mechanisms and determinations of neuronal fate during early neural development and their evolutionary interpretation. The anteroposterior patterning of the central nervous system follows an activation/transformation model, suggesting that a prospective telencephalic fate will be activated by default during the neural induction stage, with the anterior fate transformed posteriorly per caudalization morphogens. Although both extrinsic signals and intrinsic transcription factors have been implicated in dorsoventral (DV) specification of vertebrate telencephalon, the DV patterning model remains elusive. Dr. Zhang's team assumed that human forebrain DV patterning also follows an activation/transformation paradigm. Human neuroectoderm (NE) will activate forebrain dorsal fate automatically and this default anterior dorsal fate does not depend on Wnts activation or Pax6 expression. Forced expression of Pax6 in human NE hinders its ventralization, even under sonic hedgehog (Shh) treatment, suggesting that dorsal genes repress the ventral fate. Genetic manipulation of Nkx2.1, a key gene for forebrain ventral progenitors, showed that Nkx2.1 is neither necessary nor sufficient for Shh-driven ventralization. Dr. Zhang's study proposed that Shh represses dorsal genes of human NE and subsequently transforms the primitively activated dorsal fate ventrally in a repression release manner (Chi et al., 2016).

**Dr. Liang Wang** (Beijing Institute of Psychology, CAS) discussed the electrophysiological basis and function of the human brain connectome. Using standard fMRI paradigms, Dr. Wang's group identified 25 topographic maps in a large population of individual subjects and transformed them into either surface- or volume-based standardized spaces. By evaluating the topographic organization across the whole visual cortex, novel information about the organization of individual visual field maps and large-scale biases in visual field coverage were provided, with each atlas for use with independent subjects then validated. The probabilistic atlases quantified the variability of topographic representations in the human cortex, and thus provide a useful reference for comparing data across studies that can be transformed into these standard spaces (Wang et al., 2015).

**Dr. Yan-Jiang Wang** (The Third Military Medical University) discussed peripheral Aβ clearance in Alzheimer's disease (AD) brains. Dr. Wang discussed available evidence regarding the mechanisms of both endogenous and exogenous Aβ-specific antibodies, with a view to developing optimal immunotherapy based on peripheral Aβ clearance, targeting the toxic domain of Aβ, and improving antibody specificity. Such strategies could improve immunotherapy safety and efficacious disease-modifying treatment options for AD (Liu et al., 2012; Wang et al., 2016).

**Dr. Jia-Li Li** (Kunming Institute of Zoology, CAS) described recent progress in the study of epigenetic primate brains during development and aging. In their study, comprehensive RNA-Seq analysis was applied to characterize dynamics of lncRNA expression in rhesus macaque brains across postnatal development and aging. A total of 18 anatomically diverse lncRNA modules and 14 mRNA modules representing spatial, age, and sex specificities were identified. Co-expression and negative correlation between lncRNAs and mRNAs were functionally associated with brain development and aging, especially in the neocortex. These findings provide insight into spatial-, age- and sex-related dynamics of lncRNA expression during postnatal brain development and aging in macaques, implying that high dynamics of lncRNA expression might represent a previously unappreciated regulatory mechanism in shaping brain architecture and function (unpublished data).

**Drs. Jian-Hong Wang and Gong Chen** (KIZ, CAS) presented recent work on the *in vivo* reprogramming of reactive glial cells directly into the functional neurons by single neural transcription factor NeuroD1 in the monkey brain. Stroke is a major and difficult to cure brain disorder. Clinical and experimental studies have struggled to resolve glial scars after stroke. Dr. Chen has led groundbreaking work on reprogramming reactive glial cells directly into neurons in the mouse brain with injury or AD (Guo et al., 2014), leading to a novel method to reverse glial scars to neural tissue. Dr. Chen reported on the chemical reprogramming of human astrocytes into functional neurons with a cocktail of nine small molecules. This chemical reprogramming is mediated through epigenetic silencing of glial genes and transcriptional activation of neural transcription factors such as NeuroD1 and Neurogenin 2 (Zhang et al., 2015). Dr. Chen's team have also successfully established a focal cerebral ischemia model in the rhesus monkey, and successfully expressed the NeuroD1 transcription factor in the ischemic area through infection of adeno-associated virus AAV2/9-NeuroD1. They found that NeuroD1 is expressed in the monkey's cortex and protects the brain after endothelin-1 (ET-1) induced newborn neurons in NeuroD1-infected areas, paving the way for the potential clinical application of this new technology. Implementation of this study suggests a new strategy for the treatment of stroke and other brain disorders.

### Stem cell and transgenic NHP models

In the past decade, particularly in the last couple of years, the development of novel genome editing tools (ZFNs, TALENs, CRISPR/Cas9 system) has broadened the possibility of adapting mouse transgenic techniques to NHP.

**Dr. Ping Zheng** (KIZ, CAS) presented the latest study from her lab on the development of the transgenic tree shrew (*Tupaia belangeri*) model using genetically modified spermatogonial stem cells. Tree shrews have a close relationship to primates and have many advantages over rodents in biomedical research. However, a lack of gene manipulation methods has hindered their wider usage. Dr. Zheng described a culture system for the long-term expansion of tree shrew spermatogonial stem cells (SSCs) without the loss of stem cell properties. The expanded tree shrew SSCs were transfected with enhanced green fluorescent protein (eGFP)-expressing lentiviral vectors. After transplantation into sterilized adult male tree shrew testes, the eGFP-tagged SSCs could restore spermatogenesis and successfully generate transgenic offspring. Thus, the development of a culture system to expand tree shrew SSCs in combination with gene editing paves the way for precise genome manipulations using the tree shrew (Li et al., 2016).

**Dr. Yu-Yu Niu** (Kunming University of Science and Technology) discussed the development of human disease models in NHPs using gene-editing technology. Dr. Niu discussed NHPs as powerful experimental models to study neurodegenerative human diseases, such as Parkinson's, Alzheimer's, and Huntington's diseases, which occur due to genetic mutations. Because such human diseases do not occur naturally in NHPs, transgenic NHPs need to be established to understand the etiology of disease pathology and pathogenesis. Compared with rodent genetic models, the generation of transgenic NHPs for human diseases is inefficient, and only a few transgenic monkey models have been reported. Dr. Niu's group have focused on the potential approaches and contributing factors for generating transgenic NHPs to study human diseases (Chen et al., 2016a).

**Dr. Yue-Jun Chen (Shanghai Institute of Neuroscience, CAS)** presented recent work on the chemical control of grafted human pluripotent stem cell (hPSC)-derived neurons in a mouse model of Parkinson's disease (PD). The study showed tunable rescue of motor function in a mouse model of PD, following transplantation of human midbrain dopaminergic (mDA) neurons differentiated from hPSCs engineered to express designer receptors exclusively activated by designer drugs (DREADDs). Administering clozapine-N-oxide (CNO) enabled precise DREADD-dependent stimulation or inhibition of engrafted neurons, revealing D1 receptor-dependent regulation of host neuronal circuitry by engrafted cells. Transplanted cells rescued motor defects, which could be reversed or enhanced by CNO-based control of graft function, and activating engrafted cells drove behavioral changes in transplanted mice. These results highlight the ability to exogenously and noninvasively control and refine therapeutic outcomes following cell transplantation (Chen et al., 2016b). 

**Dr. Tian-Qing Li **(Kunming University of Science and Technology) described recent study on primate pluripotent stem cells and their application in brain science. Dr. Li's group established chimeric monkey animals using embryonic stem cells (ESCs) and showed that cynomolgus monkey ESCs (cESCs) grown in adjusted culture conditions could be incorporated into host embryos and develop into chimeras with contribution in all three germ layers and in germ cell progenitors. Under optimized culture conditions, which were based on an approach developed previously for naive human ESCs, the cESCs displayed altered growth properties, gene expression profiles, and self-renewal signaling pathways, suggestive of an altered naive-like cell state. These findings show that it is feasible to generate chimeric monkeys using ESCs, thus opening new avenues for the use of NHP models to study both pluripotency and human disease (Chen et al., 2015). 

**Drs. Zheng-Bo Wang and Xin-Tian Hu **(KIZ, CAS) shared their recent work on neurons differentiated from transplanted stem cells, which were shown to respond functionally to acoustic stimuli in the awake monkey brain. They developed a technique in which a small "hole" is created in the inferior colliculus (IC) of rhesus monkeys, with stem cells then transplanted *in situ* to investigate their integration into the auditory neural network. They found that some transplanted cells differentiated into mature neurons and formed synaptic input/output connections with the host neurons. They further verified that the transplanted cells have the potential to functionally integrate into the host neural network (Wei et al., 2016).

**Dr. Xu-Dong Zhao **(KIZ, CAS) reviewed the importance of primates in biomedical research, especially in preclinical research, immunology, tumor immunology, and immunotherapy. The nonhuman glioblastoma model established by Dr. ZHAO's lab showed similar histopathology, expression profiles, and image characteristics to those of human patients (unpublished data).

### NHP animal welfare and others

The three presentations in this session highlighted NHP research outside of China, as well as ethical and welfare awareness and the application of NHP resources in trans-medical research.

**Prof. Eilon Vaadia **(Hebrew University, Israel) discussed present and future direction of NHP resources and projects in Israel from the viewpoint of people, work, and outlook. Moreover, the possible collaboration for studies across multiple disciplines between KIZ and his institute were discussed. 

**Dr. De-Ming Sun **(National Health and Family Planning Commission of the PRC) introduced rules for the welfare and ethical implications of using NHP in biomedical experiments. As a representative from NHP breeding enterprises, **Mr. Zheng-Wu Wang **(Sichuan Yibin Hengshu Bio-Technology Co., Ltd.) introduced the status and market demand of nonhuman primate breeding in China, and discussed the possibility of industry-university research cooperation.

**Dr. Li Wang** (Sichuan University) introduced the urgent need for and challenges in the evaluation of drug efficacy and opportunities of NHP models in the field. Examples for the application of NHP models in human diseases, such as heart failure, neural disorders, and diabetes, in the Chengdu-based drug evaluation center, one of the largest in China, were also presented.

In summary, the organizers of the 4^th^ NHP symposium would like to take this opportunity to express our appreciation and gratitudes to all the attendees for their excellent lectures and active participation in discussions during the meeting. The organizers sincerely hope that the next symposium will continue to flourish both domestically and internationally and offer researchers many opportunities to collaborate and advance innovative research, from basic studies to translational medicine, using NHP resources.

## ACKNOWLEDGEMENTS

We are grateful to all the speakers for help in improving this manuscript. The symposium was supported by the Key Laboratory of Animal Models and Human Disease Mechanisms of the Chinese Academy of Sciences & Yunnan Province, KPRC, KIZ, CAS, and *Zoology Research*.